# US Public Health Gains from Improved Treatment of Hypercholesterolemia: A Simulation Study of NHANES Adults Treated to Guideline-Directed Therapy

**DOI:** 10.1007/s11606-025-09625-0

**Published:** 2025-06-30

**Authors:** G. Caleb Alexander, Jill Curran, Alejandro Victores, Hemalkumar B. Mehta, Shanshan Lin, Xuya Xiao, Erin D. Michos, Jeromie Ballreich, Lori D. Bash, Jason Exter, Kathryn Foti, Seth S. Martin

**Affiliations:** 1 Center for Drug Safety and Effectiveness, Johns Hopkins Bloomberg School of Public Health, Baltimore, MD USA; 2 Department of Epidemiology, Johns Hopkins Bloomberg School of Public Health, Baltimore, MD USA; 3 Division of General Internal Medicine, Johns Hopkins Medicine, Baltimore, MD USA; 4 Merck & Co., Inc., Rahway, NJ USA; 5 Division of Cardiology, Johns Hopkins School of Medicine, Baltimore, MD USA; 6 Department of Health Policy and Management, Johns Hopkins Bloomberg School of Public Health, Baltimore, MD USA; 7 Department of Epidemiology, University of Alabama at Birmingham School of Public Health, Birmingham, AL USA; 8 Ciccarone Center for the Prevention of Cardiovascular Disease, Johns Hopkins Medicine, Baltimore, MD USA

**Keywords:** Cholesterol, LDL-C, Cardiovascular disease, Treatment guidelines

## Abstract

**Importance:**

Hypercholesterolemia is widely undertreated.

**Objective:**

To project anticipated improvements in treatment and outcomes under full implementation of US and European pharmacologic treatment recommendations.

**Design, Setting, and Participants:**

The study sample included a total of 4980 adults aged 40–75 years from the 2013 through March 2020 US National Health and Nutrition Examination Survey (NHANES). We estimated the number of individuals eligible to receive versus currently receiving lipid lowering therapy (LLT) after applying: (1) the AHA/ACC guideline (“2018 US guideline”); (2) the ESC/EAS guideline (“2019 EU guideline”); and (3) the ACC expert decision pathway (“2022 US pathway”).

**Main Outcomes and Measures:**

(1) Number of individuals eligible for LLT; and (2) expected reduction in LDL-C and major cardiovascular events.

**Results:**

The study sample represented 131 million US adults. A total of 23% of the NHANES primary prevention cohort was currently using LLT compared to the 2018 US guideline/2022 US pathway (47% eligible) and the 2019 EU guideline (87% eligible). LLT use was significantly lower than the proportion of eligible patients for all therapies, including statins (66% use vs. 100% eligibility), ezetimibe (4% vs. 31–74% eligibility under the various recommendations) and proprotein convertase subtilisin/kexin type 9 inhibitors (PCSK9i) (0% vs. 11–53% eligibility). The additional overall median LDL-C reduction expected under fully guideline concordant care was 37.2 (IQR 6.7–57.6) mg/dL, 48.5 (IQR 33.0–69.9) mg/dL, and 46.8 (IQR 7.2–67.6) mg/dL based on the 2018 US guideline, 2019 EU guideline and the 2022 US pathway, respectively. These reductions in LDL-C could yield a 21–27% relative reduction in risk of major cardiovascular events.

**Conclusions and Relevance:**

Aligning treatment of hypercholesterolemia with US and European guidelines would generate major clinical and public health gains.

**Graphical Abstract:**

**Figure:**
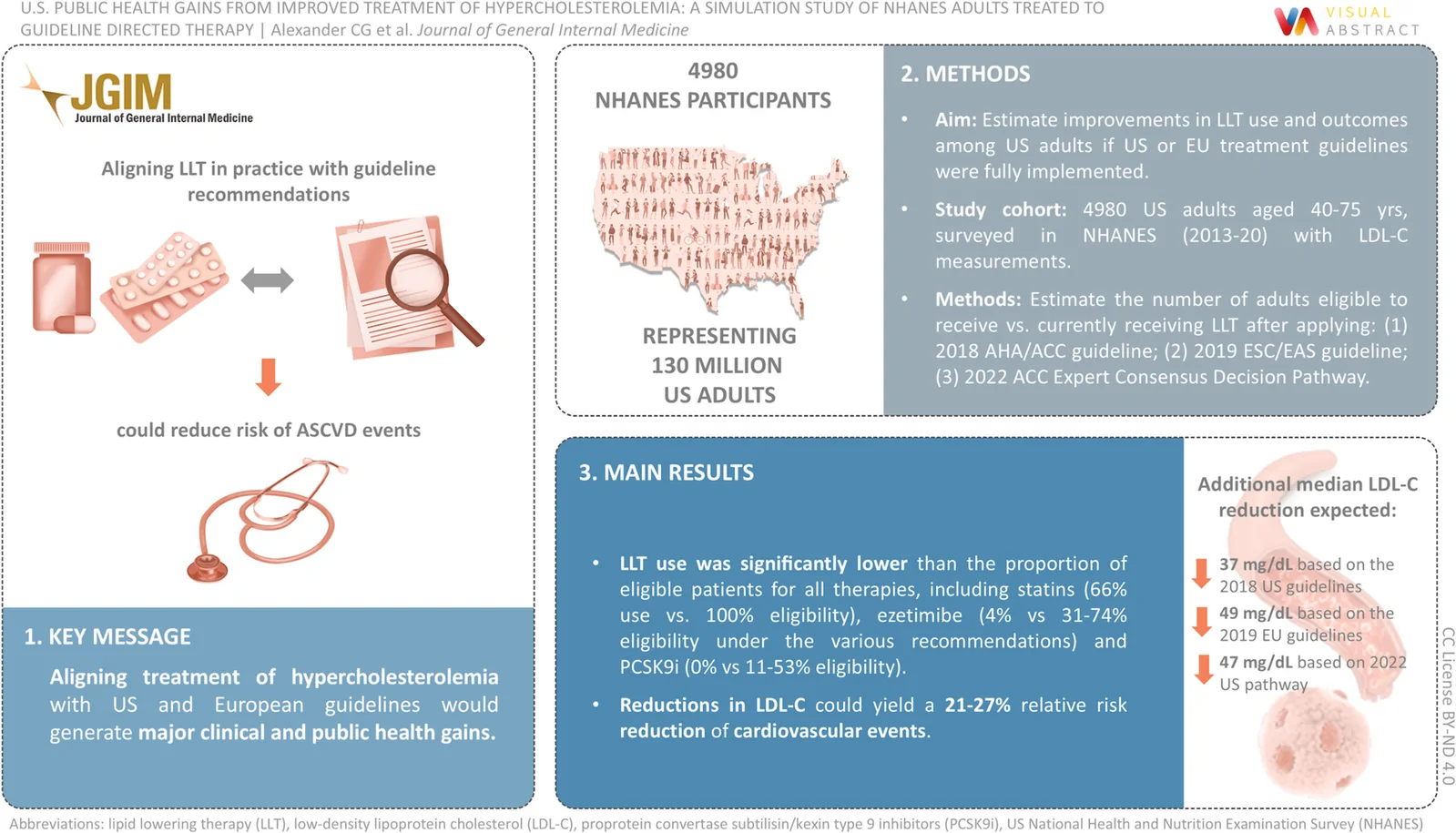

**Supplementary Information:**

The online version contains supplementary material available at https://doi.org/10.1007/s11606-025-09625-0.

## BACKGROUND

Cardiovascular disease (CVD) is the leading cause of death in the United States (US).^1,2^ While pharmacologic treatments are effective at reducing low-density lipoprotein cholesterol (LDL-C) and the risk of atherosclerotic CVD (ASCVD), there is widespread underuse of statin and non-statin lipid lowering therapies. For example, about one-quarter to one-half of individuals in the US are eligible for, but do not receive, guideline-recommended therapy for high cholesterol, with estimates varying by a given subgroup’s degree of cardiovascular risk.^3–11^

Despite insights from prior assessments quantifying undertreatment,^12–14^ these analyses leave several questions unanswered, including whether such gaps have narrowed with changes in the diagnosis and treatment of high cholesterol, as well as the magnitude of health care gains that might be expected with improved LDL-C treatment. In addition, the methods that guidelines use for risk assessment and treatment vary, and little work has been done examining the clinical and public health impact of these differences for both primary and secondary prevention.^11,15–17^

We used a nationally representative sample of the noninstitutionalized US civilian population to project potential improvements in pharmacologic treatment of lipids and consequent improvement in LDL-C and clinical cardiovascular outcomes associated with full implementation of three major recent guidelines or pathways: (1) the American Heart Association (AHA)/American College of Cardiology (ACC) guideline (“2018 US guideline”);^15^ (2) European Society of Cardiology (ESC)/European Atherosclerosis Society (EAS) guideline (“2019 EU guideline”);^18^ and (3) ACC Expert Consensus Decision Pathway on the Role of Nonstatin Therapies for LDL-Cholesterol Lowering (“2022 US pathway”).^19^ In addition to being important in their own right, our analyses may also be valuable as guidelines regarding cholesterol treatment continue to evolve.

## METHODS

### Study Design

We performed a simulation study using cross-sectional data from the US National Health and Nutrition Examination Survey (NHANES) from January 2013 through March 2020. NHANES is a nationally representative survey sampled from the US non-institutionalized civilian population conducted by the National Center for Health Statistics; it combines interviews, including demographic, socioeconomic, dietary, and health-related questions, with objective findings such as medical examinations, physiologic measurements, and laboratory tests.^20^

We used detailed information on participant demographic and clinical characteristics to estimate the extent of pharmacologic treatment of LDL-C based on US and EU recommendations separately. Then, using information regarding the clinical benefits of lipid-lowering therapy (LLT),^12,15^ we estimated the clinical gains projected to result from improved use of LLT among individuals eligible for treatment in this nationally representative population.

### Study Population

**Figure 1:**
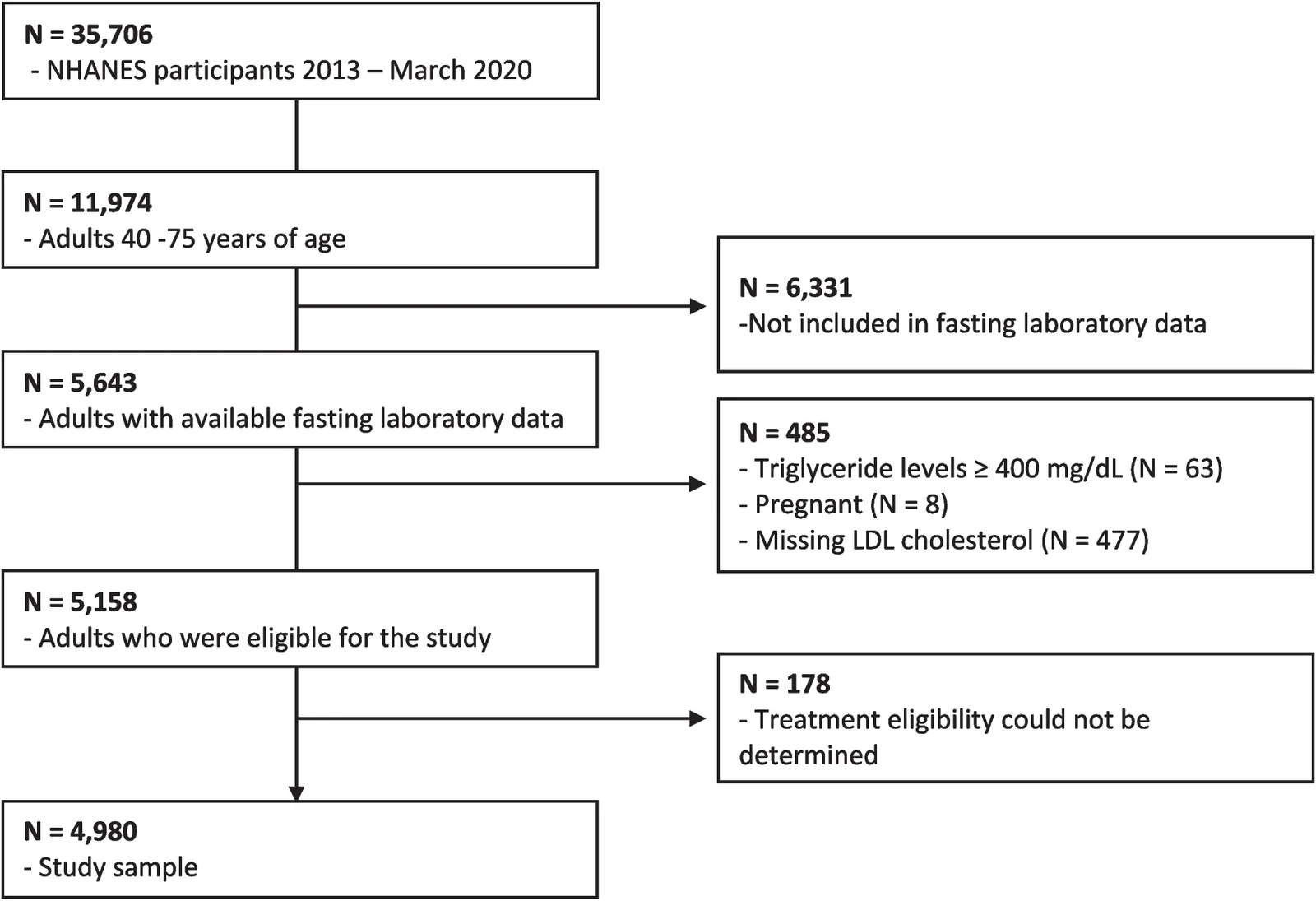
Derivation of study sample using National Health and Nutrition Examination Survey (NHANES) 2013–March 2020

We used the same cohort of adults 40–75 years old from the NHANES fasting subsample for analyses of each of the three treatment recommendations that we examined. Among 5643 adults in the fasting subsample, which included individuals eligible for both primary and secondary prevention, we excluded a total of 485 (11.7%) individuals including: individuals missing LDL-C values; individuals with triglyceride levels ≥ 400 mg/dL (given that both the Friedewald^21^ and Martin/Hopkins^22^ equations were developed and validated for patients with triglyceride levels < 400 mg/dL); and individuals who were pregnant during the survey, given that this was a contraindication for statin therapy during the time of patient data collection. While there has since been growing evidence of the benefits of statins during pregnancy,^23^ the Food and Drug Administration (FDA) label change was made in 2021,^24^ and with only eight pregnant individuals overall, we conservatively excluded them. We excluded an additional 178 individuals whose treatment eligibility could not be determined due to missing information needed for risk assessment, such as blood pressure levels or tobacco use, leaving a final study sample of 4980 adults (Fig. 1). Estimates were weighted using fasting subsample weights to generate nationally representative estimates of adults aged 40–75 years eligible for our study. We calculated weighted population totals based on recommendations from the CDC.^25^

### Estimation of Cardiovascular Risk

**Figure 2:**
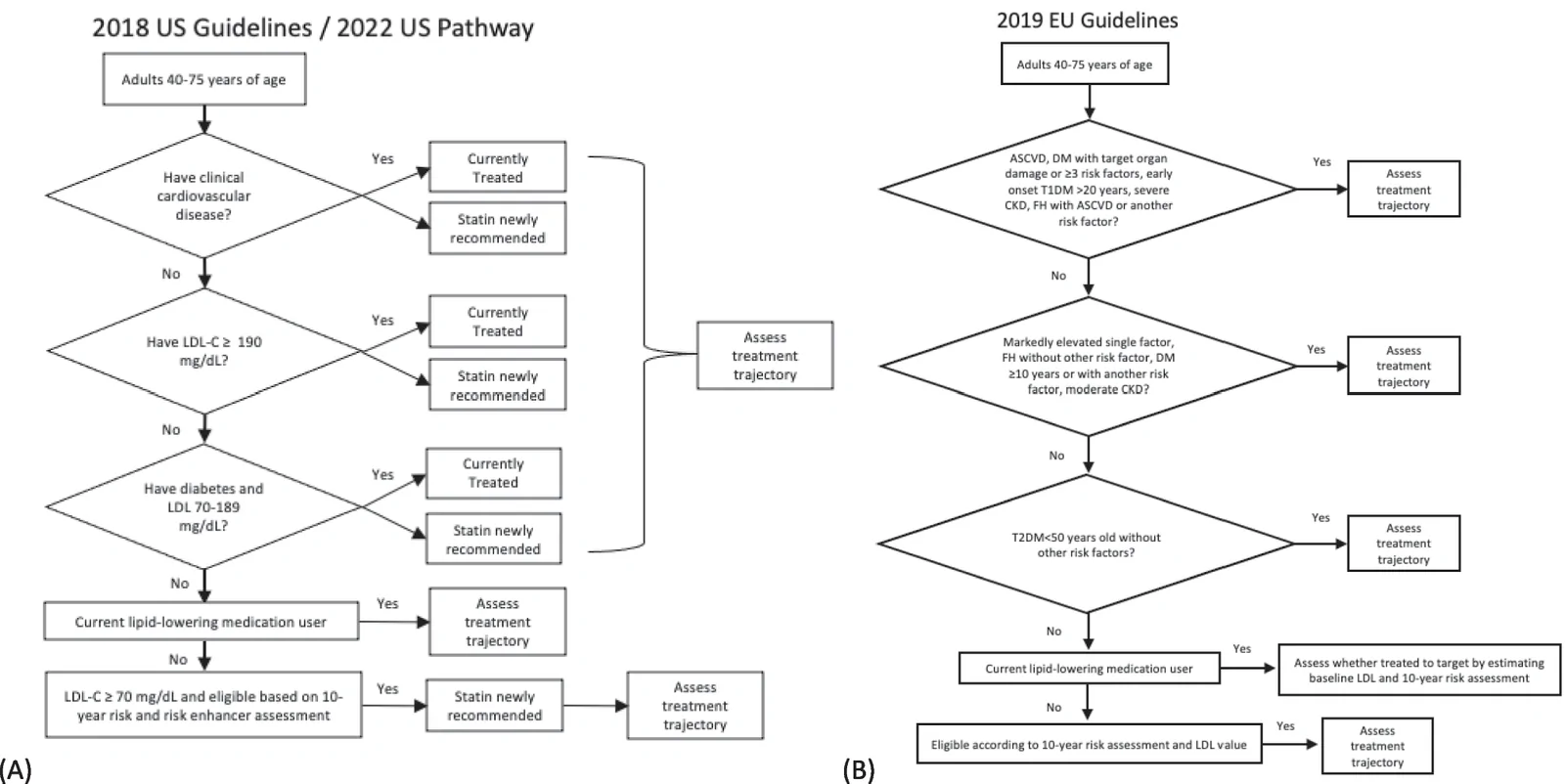
Flowchart depicting the population eligible for lipid-lowering treatment by guideline or pathway. *The 2022 US pathway uses the same flow as the 2018 US guideline with changes and additions to the treatment trajectory as described in the “Methods.” See eFigures 1–3 for specific LDL treatment thresholds.

We used a hierarchical approach to apply the 2018 US guideline (Fig. 2A). We sequentially characterized: (1) presence of clinical ASCVD, using the guideline to further stratify this group into those at very high risk or not at very high risk; (2) severe primary hypercholesterolemia (LDL-C ≥ 190 mg/dL); (3) diabetes with LDL-C 70–189 mg/dL; (4) current LLT use; and (5) LDL-C 70–189 mg/dL without diabetes or ASCVD. For this latter group, we used the Pooled Cohort Equations (PCE), endorsed by the US guidelines to predict 10-year risk of ASCVD, to further stratify individuals into four primary prevention risk groups (low (< 5%), borderline (5– < 7.5%), intermediate (7.5– < 20%), and high (≥ 20%) 10-year estimated ASCVD risk).^26^ We defined our secondary prevention cohort as individuals with clinical cardiovascular disease, including those who self-reported coronary heart disease, angina/angina pectoris, myocardial infarction, or stroke; all other individuals were considered as part of the primary prevention cohort. We used a similar process to stratify and apply the 2019 EU guideline (Fig. 2B) and the 2022 US pathway (eAppendix). NHANES does not include all necessary information to apply each set of recommendations and so we made several key assumptions based on the literature and expert input (eTables 1–3).

### Assignment to Lipid-lowering Therapy

We estimated the number of individuals in each risk group eligible for specific pharmacologic treatments, limiting pharmacologic treatments to statins, ezetimibe, and proprotein convertase subtilisin/kexin type 9 inhibitors (PCSK9i), since these three medication classes are the most widely recommended LLTs in both the US and EU guidelines. For example, the 2018 US guideline indicates that individuals with clinical ASCVD should be treated with a high-intensity statin or maximally tolerated statin. For those with clinical ASCVD, we quantified the proportion who were not receiving any LLT and assumed these individuals would be started on a high-intensity statin. We assessed their treatment trajectory from that point based on reduction in LDL-C expected with high-intensity statin treatment, and whether the individual would attain the desired LDL-C level below the threshold of 70 mg/dL or if the individual would also be eligible for additional treatment due to their risk of ASCVD.

Because the 2018 US guideline does not specify precisely how risk enhancers should guide treatment for individuals with LDL-C 70–189 mg/dL without ASCVD or diabetes, based on clinical expert opinion, we characterized individuals as eligible for pharmacologic treatment who were borderline risk plus 2 or more risk enhancers, intermediate risk plus 1 or more risk enhancers, or high risk (eTable 2). We similarly assigned individuals to treatment using the 2019 EU guideline and 2022 US pathway (eAppendix).

### Reduction in Cardiovascular Events Associated with Guideline Concordance

We used information from the Cholesterol Treatment Trialists Collaboration (CTTC) to project reductions in the risk of major ASCVD events expected with improvements in LLT based on the difference between a participant’s current and expected LDL-C after applying the relevant guideline or pathway. We assumed that for each 1 mmol/L (~ 38.67 mg/dL) reduction in LDL-C, the risk of major cardiovascular events (including coronary death, non-fatal myocardial infarction (MI), coronary revascularization, or ischemic stroke) is reduced by 22%.^27^

### Exploratory Analysis of Cardiovascular Events Averted and Economic Savings

Exploratory analyses estimated the annual number of cardiovascular events that could be averted with fully guideline- or pathway-concordant treatment among 40–75-year-olds in the US. We adjusted for the proportion of the study population we estimated to be eligible for any LLT (53% under US guideline/pathway and 88% for EU guideline) after using the expected reduction in risk of major cardiovascular events calculated for each of the three scenarios, and estimates of the annual number of events (non-fatal events MI, coronary heart disease (CHD) death, coronary revascularization, and fatal or non-fatal stroke^2^). To calculate the annual cost savings of guideline-concordant care, we used published sources of direct costs for all four major CVD events (coronary death,^28^ non-fatal MI,^29^ coronary revascularizations,^30^ and stroke^2^). We multiplied annual costs by the expected reduction in risk and inflated the cost to 2022 US dollars.

### Sensitivity Analyses

We performed several sensitivity analyses. First, we varied estimates of the proportion of current statin users at maximum tolerated dosage, which we assumed was 20% in our base case. Second, we varied the LDL-C lowering effect of each statin intensity, assuming in sensitivity analyses that this ranged from 10 to 30% for low intensity, 30 to 50% for moderate intensity, and 50 to 60% for high-intensity statins. Third, we restricted analyses to NHANES data from January 2017 to March 2020, a window where all non-statin therapies such as ezetimibe and PCSK9i were FDA approved. Finally, we performed all analyses using the Martin-Hopkins equation to estimate LDL-C, rather than Friedewald equation (which was used for main analyses), since this method of calculating LDL-C provides better accuracy, especially among individuals with low LDL-C and increased triglyceride levels.^12,31^

The data were exempt from review by an institutional review board at the Johns Hopkins Bloomberg School of Public Health. Data analyses were conducted using R (version 4.1.1, R Foundation for Statistical Computing, Vienna, Austria).

## RESULTS

A total of 4980 NHANES participants aged 40–75 years were eligible for inclusion (Fig. 1), representing 131.1 million US noninstitutionalized adults (Table 1). The median LDL-C in the weighted population was 114 mg/dL with just over one-quarter of individuals (28%) using any LLT, including statins, ezetimibe, or PCSK9i.

**Table 1:** Demographic and Clinical Characteristics of US Adults Aged 40–75 Years, National Health and Nutrition Examination Survey January 2013–March 2020 (*N* = 4980)

	Unweighted *N* = 4980	Weighted, in millions *N* = 131.1
Age, years, median (IQR)	57 (49, 65)	56 (48, 64)
Age group, *N* (%)		
40–49	1339	38.4 (29.3)
50–59	1453	41.5 (31.7)
60–69	1568	37.9 (28.9)
70–75	620	13.2 (10.1)
Female,* N* (%)	2561	67.7 (51.7)
Race and ethnicity, *N* (%)		
Non-Hispanic White	1764	88.3 (67.3)
Non-Hispanic Black	1185	14.3 (10.9)
Non-Hispanic Asian	593	7.0 (5.4)
Hispanic	1272	17.0 (12.9)
Other	166	4.5 (3.5)
Total cholesterol, mg/dL, median (IQR)	189 (162, 216)	192 (166, 219)
LDL cholesterol, mg/dL, median, (IQR)	112 (88, 136)	114 (90, 138)
HDL cholesterol, mg/dL, median, (IQR)	52 (43, 63)	52 (43, 65)
LDL cholesterol, *N* (%)		
< 70 mg/dL	494	11.4 (8.7)
70–100 mg/dL	1321	33.1 (25.2)
101–190 mg/dL	3014	82.9 (63.2)
≥ 190 mg/dL	151	3.7 (2.9)
Clinical cardiovascular disease, *N* (%)	637	14.8 (11.3)
Coronary heart disease	250	6.3 (4.8)
Angina/angina pectoris	153	3.9 (3.0)
Heart attack	274	6.2 (4.8)
Stroke	251	5.5 (4.2)
Family history of premature ASCVD, *N* (%)	23	0.6 (0.5)
Hypertension*, *N* (%)	3277	79.7 (60.8)
Diabetes^†^, *N* (%)	1072	21.7 (16.6)
Chronic kidney disease, *N* (%)	290	5.8 (4.4)
BMI, median (IQR)	28.8 (25.2, 33.7)	28.8 (25.2, 33.4)
BMI category, *N* (%)		
< 18.5 kg/m^2^	54	1.4 (1.1)
18.5–24.9 kg/m^2^	1112	28.9 (22.1)
25–29.9 kg/m^2^	1657	46.1 (35.2)
≥ 30 kg/m^2^	2107	54.7 (41.7)
Metabolic syndrome, *N* (%)	2408	61.2 (46.7)
Current smoking, *N* (%)	1009	23.4 (17.9)
Premature menopause, *N* (%)	56	4.0 (3.0)
Inflammatory disease, *N* (%)	51	1.1 (0.9)
High-sensitivity C-reactive protein ≥ 2.0 mg/L, *N* (%)	1884	65.6 (50.0)
Apolipoprotein B ≥ 130 mg/dL, *N* (%)	263	13.3 (10.2)
Use of lipid lowering therapy,^‡^ *N* (%)		
Statin use	1401	36.1 (27.6)
Ezetimibe use	40	1.5 (1.1)
PCSK9i use	2	0.05 (0.04)
Combined use^b^	30	0.9 (0.7)
Any use	1413	36.7 (28.0)

Source: National Health and Nutrition Examination Survey, 2013–March 2020

*Abbreviations*: *ASCVD*, Atherosclerotic cardiovascular; *IQR*, Interquartile range; *LDL*, Low-density lipoprotein; *HDL*, High-density lipoprotein; *PCSK9i*, Proprotein convertase subtilisin/kexin type 9 inhibitor

*Hypertension defined as SBP ≥ 130 mm Hg or DBP ≥ 80 mm Hg; self-report of a prior diagnosis of hypertension; or self-reported antihypertensive medication use

^†^Diabetes defined as participants with one or more of the following: self-report of a prior diagnosis of diabetes; fasting glucose ≥ 126 mg/dL; HbA1c ≥ 6.5%; self-reported use of insulin or oral glucose-lowering medications

^‡^Lipid-lowering therapy includes statin, ezetimibe, or PCSK9i

^b^Includes combination use of statin, ezetimibe, and/or PCSK9i

### Estimates of Lipid-Lowering Therapy Utilization and Recommendations

**Figure 3:**
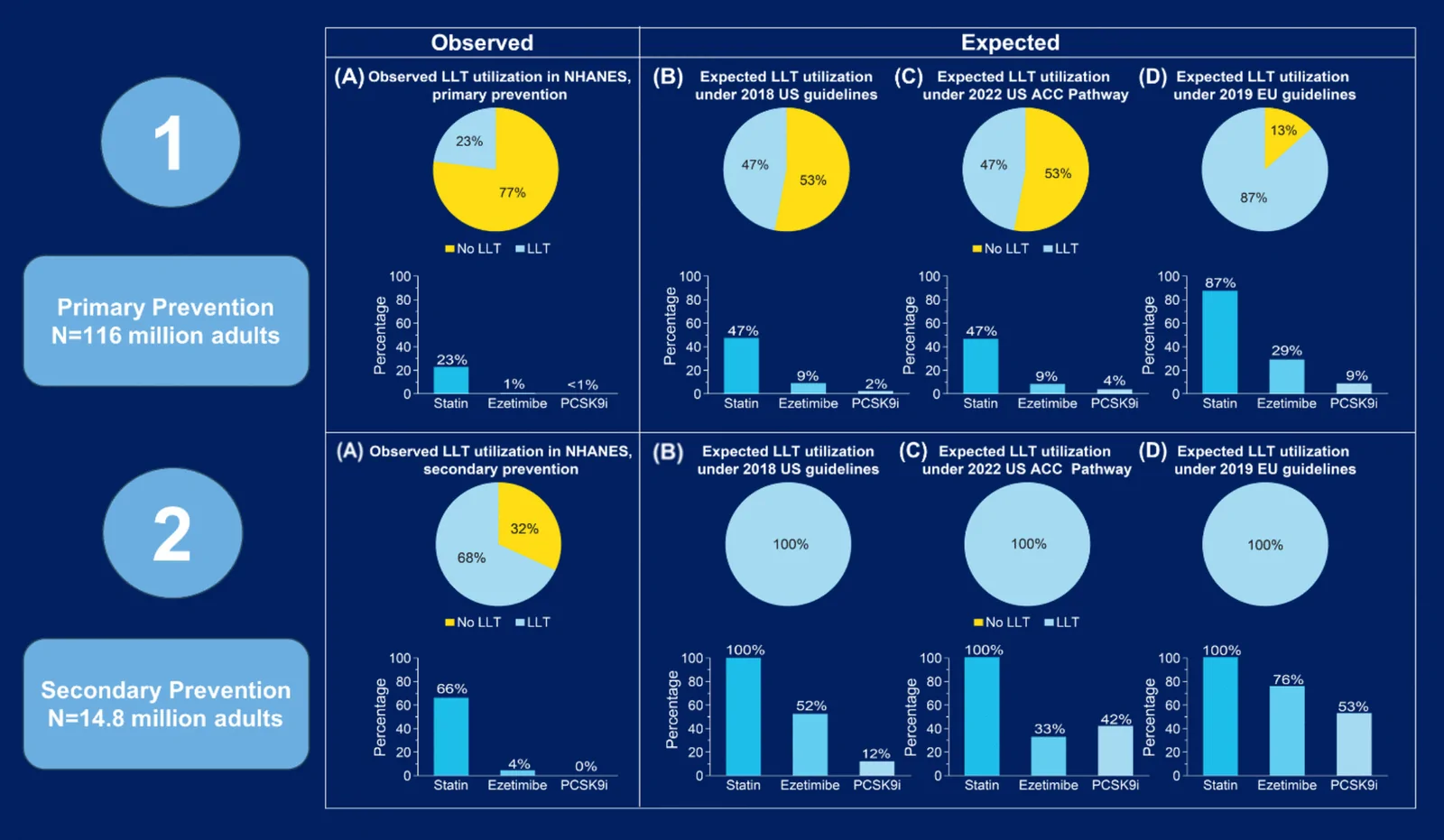
Observed versus expected^*^ guideline-concordant utilization of lipid-lowering therapies among US adults (weighted, *N* = 131.1 million individuals). ^*^Expected guideline-concordant utilization is defined as the proportion of US patients who would be prescribed each drug class under ideal conditions of fully guideline- and pathway-concordant care. ^†^Secondary prevention is defined as individuals with clinical cardiovascular disease for both the US guidelines and EU guidelines. Clinical cardiovascular disease was defined as individuals in NHANES who self-reported coronary heart disease, angina/angina pectoris, myocardial infarction, or stroke. ^‡^Represents a true zero value, there was no PCSK9i use among the secondary prevention cohort. Abbreviation: PCSK9i, proprotein convertase subtilisin/kexin type 9 inhibitor. Source: National Health and Nutrition Examination Survey, 2013–March 2020.

Figure 3A depicts use of any LLT as well as LLT use by therapeutic class observed in NHANES adults compared with expected utilization under fully guideline and pathway concordant care. Approximately 89% of patients were included in the primary prevention cohort, while 11% of patients were included in the secondary prevention cohort. Among 116.3 million adults eligible for primary prevention in NHANES, 23% received some LLT. A vast majority of these patients received statins, while very few received ezetimibe (0.7%) or PCSK9i (0.04%) therapy (Fig. 3(1A)). By contrast, many more patients were eligible for any LLT based on the 2018 US guideline (47%) (Fig. 3(1B)), 2019 EU guideline (87%) (Fig. 3(1C)), and 2022 US pathway (47%) (Fig. 3(1D)) than were receiving LLT. Eligibility for non-statin therapies varied widely across the three guidelines, with up to 9% of patients eligible for ezetimibe under the two US recommendations (Fig. 3(1B,C)) and 29% eligible under the EU guideline (Fig. 3(1D)). Similar variation was seen in PCSK9i eligibility, with 2% eligible under the 2018 US guideline (Fig. 3(1B)), 4% eligible under the 2022 US pathway (Fig. 3(1C)), and 9% eligible under the 2019 EU guideline (Fig. 3(1D)).

Among 14.8 million eligible for secondary prevention LLT according to all three recommendations, approximately 10.1 million individuals (68%) received any LLT. In addition to marked underuse of statins, non-statin therapies were underutilized for secondary prevention when applying all three recommendations. For example, 0.7 million of 14.8 million individuals (4%) received ezetimibe (Fig. 3(2A)), as compared with recommendations that 4.8–11.2 million individuals should receive such treatment (Fig. 3(2B–D)). Notably, no adults in the secondary prevention cohort received PCSK9i therapy for secondary prevention (Fig. 3(2A)), as compared with our estimation that 12% of patients under the 2018 US guideline (Fig. 3(2B)), 42% of patients under the 2022 US pathway (Fig. 3(2 C)), and 53% of patients under the 2019 EU guideline (Fig. 3(2D)) should receive this therapeutic class, representing 1.8–7.9 million US adults.

### Estimated Reduction in Blood Cholesterol and ASCVD Risk Based on Recommendations

Table 2 depicts estimated absolute reductions in LDL-C and corresponding relative risk reduction of major events expected with fully guideline and pathway concordant care among the US population and select LLT-eligible subgroups. An estimated 69.5 million individuals would be eligible for any LLT under full 2018 US guideline/2022 US pathway concordance, while 115.6 million individuals would be eligible for any LLT under the 2019 EU guideline. While the observed median baseline LDL-C of these individuals was 109 mg/dL for 2018 US guideline/2022 US pathway, and 119 for 2019 EU Guideline, guideline-concordant care would produce an estimated further 37.2 mg/dL (IQR 6.7–57.6, 2018 US), 65.3 mg/dL (2022 US Pathway), and 66 mg/dL (2019 EU Guideline) absolute reduction in LDL-C levels. The estimated expected relative reduction in the risk of major events under fully guideline-concordant care varied by was lowest for the 2018 US guideline (21% reduction), and higher for the 2022 US pathway (26%) and the 2019 EU guideline (27%). The relative risk reduction varied across different subpopulations and was greatest for those with LDL-C ≥ 190 mg/dL eligible for primary prevention (55% relative risk reduction), and least for current LLT users without diabetes or LDL-C ≥ 190 mg/dL.

**Table 2:** Estimated Absolute Reduction in LDL-C and Absolute Risk Reduction of Cardiovascular Events for Individuals Recommended Lipid-Lowering Therapy Based on Optimized Application of the (A) 2018 US Guideline, (B) 2019 EU Guideline, and (C) 2022 US Pathway

Risk group		Observed		Expected	
	*N* (millions)	Baseline LDL-C (median (mg/dL), IQR)* in NHANES	LDL-C post-treatment (median (mg/dL), IQR)	Magnitude of LDL-C reduction^†^ (median (mg/dL), IQR)	Reduction in risk of CV event^‡^, %
(A) 2018 US guideline	69.5	109 (82, 136)	73.8 (60.0, 87.6)	37.2 (6.7, 57.6)	21.3
Primary prevention	*N* = 54.7				
LDL-C ≥ 190	3.6	207 (197, 218)	86.8 (82.8, 96.0)	124.8 (98.5, 131.4)	55.2
Diabetes and LDL-C 70–189	12.9	100 (81, 124)	74.5 (65.4, 90.0)	13.5 (6.9, 46.0)	8.3
Current LLT user without diabetes or LDL-C ≥ 190	19.7	94 (72, 117)	83.2 (62.0, 89.0)	0.0 (0.0, 30.9)	0.0
LDL-C ≥ 70 and risk assessment	18.5	127 (109, 148)	75.0 (64.2, 88.2)	52.4 (44.4, 61.6)	28.6
Secondary prevention					
Clinical cardiovascular disease	14.8	91 (71, 117)	60.0 (48.9, 66.4)	30.6 (9.0, 62.5)	17.9
(B) 2019 EU Guideline	115.6	119 (96, 142)	66.0 (48.0, 86.1)	48.5 (33.0, 69.9)	26.8
Primary prevention	*N* = 100.8				
Low risk	3.2	139 (124, 149)	97.3 (86.8, 102.6)	41.7 (37.2, 44.7)	23.5
Moderate risk	31.5	129 (115, 148)	87.5 (80.5, 93.0)	38.7 (34.5, 59.2)	22.0
High risk	31.4	123 (98, 149)	61.5 (55.5, 68.0)	60.5(45.5, 88.2)	32.2
Very-high risk without ASCVD	18.7	113 (85, 142)	44.0 (26.6, 49.6)	70.2 (48.5, 118.4)	36.3
Current LLT not included in categories above	16.0	101 (82, 119)	85.0 (73.0, 90.5)	12.1 (0.0, 31.4)	7.5
Secondary prevention					
Clinical cardiovascular disease	14.8	91 (71, 117)	34.4 (26.7, 47.6)	61.4 (27.0, 79.0)	32.6
(C) 2022 US pathway	69.5	109 (82, 136)	65.3 (50.0, 83.2)	46.8 (7.2, 67.6)	26.0
Primary prevention	*N* = 54.7				
LDL-C ≥ 190	3.6	207 (197, 218)	44.4 (42.4, 95.5)	165.6 (98.5, 173.6)	65.5
Diabetes and LDL-C 70–189	12.9	100 (81, 124)	71.3 (57.2, 84.6)	34.0 (7.1, 52.9)	19.6
Current LLT user without diabetes or LDL-C ≥ 190	19.7	94 (72, 117)	83.2 (62.0, 88.9)	0 (0.0, 30.9)	0.0
LDL-C ≥ 70 and risk assessment	18.5	127 (109, 148)	67.0 (57.6, 78.8)	60.7 (51.5, 71.3)	32.3
Secondary prevention					
Clinical cardiovascular disease	14.8	91 (71, 117)	46.0 (33.6, 58.9)	51.2 (20.6, 76.9)	28.0

*Abbreviations*: *ASCVD*, Atherosclerotic cardiovascular disease; *LDL-C*, low-density lipoprotein cholesterol (mg/dL)

*Baseline LDL-C may reflect use of lipid-lowering therapy

^†^LDL-C reduction reflects application of each guideline and pathway

^‡^Relative risk reduction is based on Cholesterol Treatment Trialists’ Collaboration model as described in the “Methods”

### Estimated Number of Events Averted and Direct Economic Impact

The estimated number of events averted under guideline-concordant care is displayed in Table 3. If the population were treated according to the 2018 US guideline it is estimated that 39,196 fewer coronary deaths, 96,330 fewer non-fatal MIs, 87,559 fewer coronary revascularizations, and 65,063 fewer strokes would occur annually. The largest number of events averted was estimated under the 2019 EU guideline, including 82,239 fewer coronary deaths, 199,689 fewer non-fatal MIs, 182,147 fewer coronary revascularizations, and 137,414 fewer strokes, with the number of events averted according to the 2022 US pathways fell between the two (Table 3). The estimated overall additional reduction in relative risk of major CVD events under the 2018 US Guideline, 2019 EU Guideline, and 2022 US Pathway would translate to estimated $25.3, $31.7, and $30.6 billion reductions in annual direct health care expenditures, respectively (eTable 4).

**Table 3:** Expected Major Cardiovascular Events Averted Under Fully Guideline-Concordant Care

	2018 US guideline	2019 EU guideline	2022 US pathway
Number avoided*			
Coronary death	39,196	82,239	48,018
Non-fatal myocardial infarction	96,330	199,689	116,805
Coronary revascularization	87,559	182,147	106,489
Stroke	65,063	137,414	80,155

*Annual event numbers in the US derived from the American Heart Association Report on heart disease and stroke statistics in 2023

### Sensitivity Analyses

The four sensitivity analyses described in the supplementary appendix yielded substantively similar quantitative findings as the primary analysis (eTable 5). An analysis of the main results depicted in Fig. 3 stratified by race is provided in eTable 6. There were modest (and larger) differences in observed utilization of LLT for primary (and secondary) prevention by race, with lowest utilization by Hispanics and highest utilization by Non-Hispanic Whites. Expected utilization by race was much more comparable for primary prevention, and identical for all secondary prevention groups.

## DISCUSSION

We used a weighted nationally representative sample of US adults to estimate the observed utilization of lipid-lowering therapy and the utilization that would be expected under three major clinical treatment recommendations. We estimate that fully guideline-concordant care would correspond to an additional 32.8 to 78.9 million US adults treated with LLT, yielding an approximate 21–27% relative reduction in the risk of major ASCVD events in the US among these individuals. Our work provides important updates to prior models of cholesterol guideline adoption,^32,33^ while simulating treatment for a nationally representative sample of 40–75-year-old US adults eligible for lipid-lowering therapy.

Our findings have several implications. First, by demonstrating the large and persistent gaps between treatment recommendations and clinical practice, our results underscore the continued importance of systematic interventions to improve quality of hypercholesterolemia treatment for individuals with elevated LDL-C.^34^ We also quantified the relative impact of different guidelines on reducing treatment gaps and rates of CVD, overall and among specific subpopulations of individuals eligible for primary and secondary prevention. We estimated the largest LDL-C reduction and corresponding reduction in risk of CV events for individuals with LDL-C of 190 mg/dL or higher without diabetes and with the 2018 US guideline and 2022 US Pathway recommendation. While these reductions in risk were high (55% and 65% for the US guideline and pathway, respectively), it should be noted that this group represented the smallest population of our risk groups identified (~ 5%). For each guideline and pathway, we observed the smallest reduction in LDL-C and risk of ASCVD events for individuals without clinical ASCVD, diabetes, or primary hypercholesterolemia, who are currently being treated with LLT, a reflection of the fact that these individuals are on LLT but at lower baseline risk of cardiovascular events compared to other treatment-eligible groups.

We observed a much larger percentage of the primary prevention population eligible for statin therapy based on the 2019 EU guideline than what was estimated with either the updated 2022 US pathway and the 2018 US guideline. This could be due in part to European guidelines’ more aggressive LDL-C targets compared to US guidelines. As expected, non-statin therapies for additional LDL-C reduction were more frequently recommended in the secondary prevention population than in the primary prevention population. Across both the primary and secondary prevention cohorts, the 2019 EU guidelines most often recommended both ezetimibe and PCSK9i. This was followed by the 2022 US pathway which recommended PCSK9i next most often in both cohorts. Interestingly, ezetimibe was recommended equally by the 2022 US pathway and the 2019 US guideline in primary prevention, and more often by the 2018 US guidelines among the secondary prevention cohort.

Our analysis identified a clear implementation gap of LLT among treatment-eligible patients. There are a variety of reasons for these gaps,^35^ including discordance between the guidelines and pathways we examined and other guidelines and algorithms,^36^ including those produced by the United States Preventive Services Task Force (USPSTF),^37^ National Committee for Quality Assurance (NCQA),^38^ and Centers for Medicare and Medicaid Services (CMS).^39^ That said, when assessing performance of various treatment recommendations in reducing LDL-C and improving CV outcomes, our findings suggest that lower risk-based LDL-C thresholds may yield the greatest benefit among US adults. In addition to increasing the use of statins, achieving protective cholesterol treatment targets will require better implementation of non-statin LLT among eligible patients. Adoption of guideline-directed LLT could not only yield effective LDL-C reductions across a growing population of at-risk adults, but has the potential to prevent about 248,000–519,000 non-fatal ASCVD events and 39,000–82,000 coronary deaths a year, translating to a total cost savings of $25.3–31.7 billion nationally.

A strength of this analysis is the use of NHANES, which is a large, nationally representative survey including demographic, laboratory, and medication information. However, limitations inherent to NHANES include the cross-sectional nature of the data, the small sample sizes among a few subgroups analyzed, self-reported information including factors such as history of CVD and medication usage, and the absence of some information that would be helpful to simulate treatment and estimate risk. Our unweighted sample included small numbers of individuals on non-statin therapies, and our data extends through March 2020; treatment patterns may have changed since then, both due to the COVID-19 pandemic as well as broader secular trends and approval of new treatments such as bempedoic acid and inclisiran. As per the US guidelines we studied, we used the PCE to predict 10-year risk of ASCVD; the PREVENT equations or other methods of estimating cardiovascular risk may yield different estimates regarding the magnitude of undertreatment of hyperlipidemia in the United States.^40^ While our treatment simulations required a variety of scientific assumptions, we were conservative in our estimates and our results were substantively similar in a variety of sensitivity analyses. Finally, our exploratory analysis evaluating the potential number of events averted through guideline concordant care includes estimates of ASCVD outcomes for individuals of all ages. While we focused on the proportion of individuals estimated to be eligible for LLT, our analyses were limited to 40–75-year-olds. However, large observational studies suggest older adults would experience a similar clinical benefit through the reduction of LDL-C.^41,42^

## CONCLUSIONS

We projected improvements in LLT utilization and cardiovascular outcomes associated with full implementation of US and European treatment recommendations for the management of LDL-C. In addition to documenting widespread and continued underuse of LLTs including statins, ezetimibe, and PCSK9i, we also demonstrate the remarkable clinical and public health gains that could be achieved with fully guideline-concordant care.

## Supplementary Information

Below is the link to the electronic supplementary material.

Supplementary file1 (DOCX 471 KB)
