# Supplementary material for: US Public Health Gains from Improved Treatment of Hypercholesterolemia: A Simulation Study of NHANES Adults Treated to Guideline-Directed Therapy
Source: J Gen Intern Med. 2025 Jun 30;41(12):3488–98. doi: 10.1007/s11606-025-09625-0 (PMC13569724; doi:10.1007/s11606-025-09625-0)
Supplement: Supplementary file 1 [file 11606_2025_9625_MOESM1_ESM.docx]

**eAppendix**

## Estimation of Cardiovascular Risk

The 2018 US guideline also recommends considering risk enhancers to inform LLT treatment for the primary prevention population at borderline or intermediate risk (**eTable 1**). Individuals with clinical ASCVD were further categorized as being secondary prevention “at very high risk” and secondary prevention “not at very high risk”. Very high risk ASCVD includes patients with two or more major cardiovascular clinical events or one event with two or more high risk features (**eTable 2**).

For the 2019 EU guidelines, we used a hierarchal approach similar to the 2018 US guidelines and sequentially classified individuals into: (1) very-high risk defined as clinical CVD, diabetes with 3 or more risk factors, severe chronic kidney disease (CKD), Familial Hypercholesterolemia (FH) with clinical CVD or another major risk factor; (2) high-risk defined as markedly elevated single factor, FH without other risk factor, diabetes with another risk factor, moderate CKD; (3) Type 2 diabetes <50 years old without other risk factor; (4) current LLT use; (5) remaining eligible participants based on 10-year risk assessment. For this latter group, we used the Systematic COronary Risk Evaluation 2 (SCORE2) risk prediction model, endorsed by the EU guideline to further stratify individuals into one of four risk groups based on 10-year risk of fatal CVD.^[1],^^[2]^ The SCORE2 uses risk regions to classify countries as low, moderate, high or very-high risk based on CVD mortality rates. Since the CVD mortality rate in the US is greater than 200 per 100,000 individuals,^[3]^ we used the high-risk model.

For the 2022 US pathway, we followed the same hierarchical approach as the 2018 US guideline, since the pathway follows the same treatment trajectory, but provides augmented clinical guidance on appropriate utilization of non-statin therapy among individuals already on statins. More detailed treatment pathways are illustrated in **eFigures 1-3**.

**Assignment to Lipid-lowering Therapy**

Using the 2018 US Guideline, for individuals with clinical ASCVD not at very high risk who were already receiving LLT and not at an LDL-C <70 mg/dL, we conservatively assumed 20% of individuals were already taking a high intensity statin and should have ezetimibe added and 80% were not at maximum dose and should have statin increased from moderate to high intensity prior to adding any non-statin therapy.^[4],^^[5]^ For the very high-risk individuals with clinical ASCVD, we followed the same progression except the 2018 US guideline recommends PCSK9i therapy if the threshold of LDL-C <70 mg/dL is not reached with high intensity statin plus ezetimibe therapy. For individuals already using LLT who did not have clinical ASCVD, severe primary hypercholesterolemia, or diabetes, we again assumed 20% of the statin users were already at a high intensity while 80% would need to be escalated from a moderate to high intensity statin. We modeled treatment for this group all the way to maximum statin plus ezetimibe plus PCSK9i until individuals attained LDL-C <100 mg/dL.

We assigned individuals to treatment using the 2019 EU guideline and 2022 US pathway similarly to the 2018 US guideline methodology. In contrast to the 2018 US guideline, both of these recommendations utilize LDL-C targets in all groups in order to guide treatment intensification. For example, the 2019 EU guideline suggests individuals with clinical CVD, classified as very high risk, should be treated to a target LDL-C <55mg/dL. The 2022 US pathway suggests individuals with LDL-C 70-189 mg/dL without diabetes who are determined to be at high risk based on an estimated PCE risk ≥20% should be treated to a target LDL-C <70 mg/dL.

As with estimations of cardiovascular risk, our approach to assigning people to specific LLT required key scientific assumptions. For example, NHANES does not include information regarding statin dosage and therefore we cannot assess what proportion of individuals already on a statin are taking the maximum tolerated dosage. We describe these assumptions in **eTable 3**, and we assessed the sensitivity of our findings and their substantive interpretation to these as described below.

**eTable 1.** Variables necessary for applying risk score calculation (used pooled cohort equations for US and SCORE2 for EU) and assessing risk enhancers in relevant clinical practice guidelines.

| Risk Factor | 2018 US guideline/ 2022 US pathway | 2019 EU guideline | Available in NHANES |
| --- | --- | --- | --- |
| Age | √ | √ | √ |
| Sex | √ | √ | √ |
| Race | √ |  | √ |
| Total cholesterol | √ | √ | √ |
| HDL cholesterol | √ | √ | √ |
| LDL cholesterol |  | √ | √ |
| Systolic blood pressure | √ | √ | √ |
| Diabetes status | √ | √ | √ |
| Blood pressure lowering medication use | √ |  | √ |
| Smoking status | √ | √ | √ |
| Family history of premature ASCVD^*^ | √ |  | √ |
| Persistently elevated LDL-C≥160 mg/dL^*^ | √ |  |  |
| Chronic kidney disease (eGFR 15-59 mL/min/1.73m^2^)^*^ | √ |  | √ |
| Metabolic syndrome ^*†^ | √ |  | √ |
| Premature menopause^*^ | √ |  | √ |
| Inflammatory disease^*^ | √ |  | √ |
| High-sensitivity C-reactive protein ≥2.0 mg/L ^*^ | √ |  | √ |
| Apolipoprotein B ≥130 mg/dL^*^ | √ |  | √ |
| Ankle-brachial index <0.9^*^ | √ |  |  |
| Lipoprotein(a) levels ≥50 mg/dL or ≥125 nmol/L^*^ | √ |  |  |
| High-risk ethnicities^*^ | √ |  |  |
| Persistently elevated triglycerides^*^ | √ |  |  |

^*^ASCVD risk enhancers according to the US guideline/pathway

^†^Metabolic syndrome was defined as at least three of the following: waist circumference>102 cm in men or 88

cm in women, triglyceride>150 mg/dL, HDL-C<40 mg/dL in men or <50 mg/dL in women, systolic blood pressure≥130 mm Hg or diastolic blood pressure≥85 mm Hg or taking hypertension medications, or fasting plasma glucose level≥100 mg/dL or taking diabetes medications

**ASCVD** Atherosclerotic cardiovascular disease **eGFR** estimated glomerular filtration rate **LDL-C** low-density lipoprotein cholesterol (mg/dL)

**eTable 2.** Variable definitions used in analyses.

| **Variables** | **Definitions** |
| --- | --- |
| *Eligibility* | |
| Age, years | Participant age in years. We included participants aged 40 to 75 years old. |
| Triglyceride, mg/dL | We excluded people with triglyceride levels ≥ 400 mg/dL. |
| Pregnant | Pregnancy status. This is defined by either self-report of being pregnant, or a positive lab pregnancy test conducted during the study visit among females. We excluded people who were pregnant during survey. |
| *Demographic characteristics* | |
| Sex | Self-reported sex (male, female). |
| Race and ethnicity | Self-reported race/ethnicity. Participants were categorized into groups: non-Hispanic White, non-Hispanic Black, non-Hispanic Asian, Hispanic (including Mexican American and other Hispanic) and other (including multi-racial). |
| *Lab results* | |
| Total cholesterol, mg/dL | The total cholesterol in mg/dL. |
| LDL-Cholesterol, mg/dL | LDL- Cholesterol is calculated using Friedewald equation, in mg/dL. |
| HDL-Cholesterol, mg/dL | Direct HDL-Cholesterol in mg/dL. |
| *Blood pressure* | |
| Systolic blood pressure (SBP), mm Hg | Mean SBP in mm Hg. In 2017-2020, systolic blood pressure was measured using an oscillometric device. The SBP in 2017-2020 was calibrated to the mercury device by adding 1.5 mm Hg to the mean measured oscillometric value. The mean SBP was determined based on available (up to 3) SBP readings. |
| Diastolic blood pressure (DBP), mm Hg | Mean DBP in mm Hg. In 2017-2020, diastolic blood pressure was measured using an oscillometric device. The DBP in 2017-2020 was calibrated to the mercury device by subtracting 1.3 mm Hg from the mean measured oscillometric value. The mean DBP was determined based on available (up to 3) DBP readings. |
| *Comorbidities* | |
| Smoking status | Self-reported current cigarette smoking. |
| Body mass index, kg/m^2^ | Body mass index in kg/m^2^, estimated using the height and weight measured during the study examination. |
| Body mass index, category, kg/m^2^ | BMI grouping: <18.5, 18.5-24.9, 25-29.9, $\geq$30 kg/m^2^. |
| History of atherosclerotic cardiovascular disease (ASCVD) | Self-reported history of coronary heart disease, myocardial infarction, stroke or angina pectoris. |
| History of myocardial infarction | Self-reported history of heart attack. |
| History of coronary heart disease | Self-reported history of coronary heart disease. |
| History of stroke | Self-reported history of stroke. |
| History of angina/angina pectoris | Self-reported history of angina pectoris. |
| Hypertension | SBP ≥ 130 mm Hg or DBP ≥ 80 mm Hg; self-report of a prior diagnosis of hypertension; or self-reported antihypertensive medication use. |
| Diabetes | Participants have any or more of the following: self-report of a prior diagnosis of diabetes; fasting glucose $\geq$126 mg/dL; HbA1c $\geq$ 6.5%; self-reported use of insulin or oral glucose-lowering medications. |
| Chronic kidney disease (the US guideline) | Estimated glomerular filtration rate $\geq$ 15 and $\leq$ 59 ml/min/1.73 m^2^. Estimated glomerular filtration rate was calculated using the 2021 serum creatinine-based equation (2009 CKD-EPI creatinine fit without race). |
| Chronic kidney disease (the EU guideline) | Severe CKD is defined as eGFR < 30 ml/min/1.73 m^2^; Moderate CKD is defined as eGFR $\geq$ 30 and $\leq$ 59 ml/min/1.73 m^2^. Estimated glomerular filtration rate was calculated using the 2021 serum creatinine-based equation. |
| Heterozygous familial hypercholesterolemia (FH) | We used LDL-C ≥ 190 mg/dL and a family history of premature ASCVD as a proxy measure |
| *Antihypertensive medication* | |
| Self-reported antihypertensive medication use | Self-reported current use of antihypertensive medication with a prior diagnosis of hypertension. |
| *Lipid lowering medication* | |
| Statin | RXDDCI1A = 358, RXDDCI1B = 19, RXDDCI1C = 173 (category name: HMG-COA REDUCTASE INHIBITORS (STATINS)) and RXDDCI1C = 317 (category name: ANTIHYPERLIPIDEMIC COMBINATIONS including all drug combinations for this variable: Lovastatin; Niacin, Ezetimibe; Simvastatin, Niacin; Simvastatin, and Simvastatin; Sitagliptin). |
| Ezetimibe | RXDDCI1A = 358, RXDDCI1B = 19, RXDDCI1C = 316 (category name: CHOLESTEROL ABSORPTION INHIBITORS) and RXDDCI1C = 317 (category name: ANTIHYPERLIPIDEMIC COMBINATIONS) and only drug combination of Ezetimibe; Simvastatin. |
| PCSK9 | RXDDCI1A = 358, RXDDCI1B = 19, RXDDCI1C = 484 (category name: PCSK9 INHIBITORS) |
| Combination use | Individuals with at least two of the above medications (not including RXDDCI1C = 173 when participants were on multiple drugs in the same class) |
| Any lipid lowering therapy use | Individuals with any medication usage including statin, ezetimibe, or PCKS9i as defined above. |
| *US guideline/pathway-specific definition* | |
| Primary prevention low risk | Calculated PCE<5% |
| Primary prevention borderline risk | Calculated PCE 5% - <7.5% plus 2 or more risk enhancers |
| Primary prevention intermediate | Calculated PCE ≥7.5% - 20% plus 1 or more risk enhancer |
| Primary prevention high risk | Calculated PCE ≥20% |
| Major ASCVD event | Major ASCVD event is defined as having history of MI or history of stroke. Other events listed in the guideline, such as recent acute coronary syndrome and symptomatic peripheral arterial disease, are not assessed in the NHANES. |
| High-risk conditions | These include: (1) age $\geq$ 65 years old; (2) Heterozygous familial hypercholesterolemia; (3) Diabetes mellitus; (4) Hypertension; (5) CKD (eGFR 15-59 ml/min/1.73 m^2^); (6) current smoking; (7) history of heart failure. |
| Very high-risk ASCVD | The very high-risk ASCVD include a history of multiple ($\geq$2) major ASCVD events or 1 major ASCVD event and multiple ($\geq$2) high-risk conditions. |
| ASCVD risk enhancers | These include: (1) family history of premature ASCVD; (2) chronic kidney disease; (3) metabolic syndrome; (4) premature menopause; (5) Inflammatory disease (especially rheumatoid arthritis, psoriasis, HIV); (6) hs-CRP $\geq$2.0 mg/L; (7) apoB $\geq$130 mg/dL. Risk enhancers of persistently elevated LDL-C, persistently elevated triglycerides, Lp(a), ankle-brachial index and ethnicity of South Asian ancestry are either not available in NHANES or not available during the included cycles. |
| History of heart failure | Self-reported history of heart failure. |
| Family history of premature ASCVD | Self-reported medical history of a heart attack in a close relative. |
| Metabolic syndrome | Participants have at least 3 of the following: (1) waist circumference greater than 102 cm in men or 88 cm in women; (2) triglyceride level greater than 150 mg/dL; (3) high-density lipoprotein cholesterol less than 40 mg/dL in men or less than 50 mg/dL in women; (4) systolic blood pressure at least 130 mm Hg or diastolic blood pressure at least 80 mm Hg or taking hypertension medications; (5) or fasting plasma glucose level at least 100 mg/dL or taking diabetes medications. |
| Premature menopause | Self-reported reason not having regular periods as “Menopause/Change of life”, having final menstruation before the age of 40 years among women aged 45 or over. |
| Rheumatoid arthritis | Self-reported history of rheumatoid arthritis. |
| Psoriasis | Self-reported history of psoriasis, only available during 2013-2014 cycle. |
| HIV | Positive HIV antibody testing result during 2013-2014 cycle, or positive HIV-1 antibody blood assay test results during 2015-2016 cycle. HIV cannot be evaluated in the 2017-March 2020 cycle. |
| Hs-CRP, mg/L | The high-sensitivity C-reactive protein (hs-CRP) in mg/L. Not available during 2013-2014 cycle. |
| ApoB, mg/dL | The apolipoprotein (B) in mg/dL. Not available during 2017-March 2020 cycle. |
| *EU guideline-specific definition* | |
| Very-high-risk | Calculated SCORE $\geq$10%; Documented ASCVD, diabetes mellitus with target organ damage (microalbuminuria) or with $\geq$3 risk factors (current smoker, dyslipidemia, hypertension, obesity), early onset of T1DM (>20 years), severe CKD, FH with ASCVD or another risk factor (current smoker, diabetes mellitus, hypertension, obesity) |
| High-risk | Calculated SCORE $\geq$ 5% and < 10%; Markedly elevated single factor (TC >8 mmol/L (>310 mg/dL), LDL-C >4.9 mmol/L (>190 mg/dL), or BP $\geq$ 180/110 mmHg), FH without other risk factor or DM without target organ damage(microalbuminuria), DM ≥10 years or with another risk factor, moderate CKD |
| Moderate-risk | Calculated SCORE $\geq$ 1% and < 5%; Young patients (T1DM < 35 years, T2DM < 50 years) with DM duration < 10 years without other risk factors |
| Low-risk | Calculated SCORE <1%. |
| Microalbuminuria | Microalbuminuria is defined as a urinary albumin-to-creatinine ratio (ACR) of ≥30 mg/G. |
| Dyslipidemia | Dyslipidemia is defined as any one or more of the following: total cholesterol ≥240mg/dL (≥6.2mmol/L) or self-reported history of having high cholesterol; LDL-C ≥160mg/dL (≥4.1mmol/L); HDL-C <40mg/dL (<1.0mmol/L); triglyceride >200mg/dL (>2.3mmol/L). |
| Type I diabetes | We defined anyone with diabetes diagnosed before the age of 30 years who was taking only insulin therapy as having type I diabetes mellitus. |
| Obesity | This is defined as BMI $\geq$30 kg/m^2^. |

**eTable 3.** Key scientific assumptions undertaken to simulate expected reductions in LDL-C and cardiovascular events among US adults under ideal application of current treatment recommendations.

| **Question** | **Relevant guideline or pathway** | **Reason assumption is needed** | **Scientific assumption** |
| --- | --- | --- | --- |
| What proportion of individuals are on maximally tolerated statin dose? | 2018 US, 2019 EU, 2022 US | NHANES lacks information on statin dose | Estimate random 20% of statin users at maximum dose, remainder should have dose escalated to guideline recommended statin prior to consideration of non-statin therapy^[6],^^[7]^ |
| What is the expected effect of statin initiation on LDL-C? | 2018 US, 2019 EU, 2022 US | To model LDL reduction expected with statin treatment | Estimate LDL-C lowering as 30% with low intensity statins, 40% with moderate intensity statins, and 50% with high intensity statins^[8],^^[9]^ |
| What is the expected effect of ezetimibe initiation on LDL-C? | 2018 US, 2019 EU, 2022 US | To model LDL reduction expected with ezetimibe treatment | Estimate LDL-C lowering as additional 20% when added to highest statin^[10],^^[11]^ |
| What is the expected effect of PCSK9i initiation on LDL-C? | 2018 US, 2019 EU, 2022 US | To model LDL reduction expected with PCSK9i treatment | Estimate LDL-C lowering as additional 60% when added to both highest tolerated statin alone or highest tolerated statin plus ezetimibe^[12],^^[13]^ |
| ,What proportion of individuals have heterozygous FH? | 2019 EU | NHANES lack information on heterozygous FH diagnosis | Defined as any individual with LDL-C≥190 mg/dL and a family history of early ASCVD^[14]^ |

Other risk factors could not be evaluated including: history of prior coronary bypass surgery or percutaneous coronary intervention outside of major ASCVD event; persistently elevated LDL-C; recent acute coronary syndrome; target organ damage; information regarding imaging, coronary revascularization, TIA; patients with ASCVD who experience second vascular event within 2 years while taking maximally tolerated statin

**Abbreviations: ASCVD** atherosclerotic cardiovascular disease **FH** familial hypercholesterolemia **LDL-C** low-density lipoprotein cholesterol **NHANES** National Health and Nutrition Examination Survey **TIA** transient ischemic attack

**eTable 4. Expected economic savings on major cardiovascular events under fully guideline-concordant care (in billions of 2022 US dollars).**

|  | 2018 US Guideline | 2019 EU Guideline | 2022 US Pathway |
| --- | --- | --- | --- |
| Number avoided |  |  |  |
| Coronary death | 3.3 | 4.1 | 4.0 |
| Non-fatal myocardial infarction | 9.3 | 11.6 | 11.2 |
| Coronary revascularization | 6.3 | 7.9 | 7.6 |
| Stroke | 6.4 | 8.1 | 7.8 |
| Total (US dollars) | 25.3 | 31.7 | 30.6 |

**eTable 5.** Results of sensitivity analyses adjusting various assumptions compared to primary analyses.

|  | **2018 US Guideline** | **2019 EU Guideline** | **2022 US Pathway** |
| --- | --- | --- | --- |
|  | **LDL-C reduction (mg/dL)** | | |
| Primary Analyses | 37.2 | 48.5 | 46.8 |
| Proportion of NHANES statin users at maximum dosage to primary analyses | | | |
| 30/70% | 38.0 | 50.0 | 47.5 |
| 40/60% | 38.7 | 50.0 | 47.4 |
| 50/50% | 38.8 | 50.0 | 47.5 |
| LDL-C lowering effect of each statin medication^*^ | | | |
| Sensitivity-Min | 31.2 | 47.1 | 53.2 |
| Sensitivity-Max | 46.4 | 68.3 | 60.0 |
| 2017-2020 data | 36.4 | 48.0 | 46.0 |
| Martin-Hopkins | 39.4 | 50.2 | 48.1 |
|  | **Reduction in risk of CV events** | | |
| Primary Analyses | 21.3% | 26.8% | 26.0% |
| Proportion of NHANES statin users at maximum dosage to primary analyses | | | |
| 30/70% | 21.7% | 27.5% | 26.3% |
| 40/60% | 22.0% | 27.5% | 26.3% |
| 50/50% | 22.1% | 27.5% | 26.3% |
| LDL-C lowering effect of each statin medication^*^ | | | |
| Sensitivity-Min | 18.2% | 26.1% | 29.0% |
| Sensitivity-Max | 25.8% | 35.5% | 32.0% |
| 2017-2020 data | 20.0% | 26.5% | 25.1% |
| Martin-Hopkins | 22.4% | 27.6% | 26.6% |

^*^For the 2018 US guideline, the combination of assumptions leading to the lowest LDL-C reduction (Sensitivity-Min) were 10% for low intensity, 30% for moderate, and 55% for high intensity statin. The highest LDL-C reduction (Sensitivity-Max) was a result of 10% for low intensity, 49.9% for moderate, and 50% for high intensity statin

For the 2019 EU guideline, Sensitivity-Min corresponded to 30% for low intensity, 30% for moderate, and 50% for high intensity statin. Sensitivity-Max was a result of 15% for low intensity, 49.9% for moderate, and 60% for high intensity statin

For the 2022 US pathway, Sensitivity-Min corresponded to 10% for low intensity, 40% for moderate, and 50% for high intensity statin. Sensitivity-Max was a result of 10% for low intensity, 49.9% for moderate, and 50% for high intensity statin

**eTable 6.** Observed versus expected guideline-concordant utilization of lipid lowering therapies among US adults, stratified by race.

|  | Non-Hispanic White  N = 88.3* | Non-Hispanic Black  N = 14.3 | Non-Hispanic Asian  N = 7.0 | Hispanic  N = 17.0 |
| --- | --- | --- | --- | --- |
| **Primary Prevention** | N = 78.3 | N = 12.4 | N = 6.6 | N = 15.7 |
| Observed LLT utilization in NHANES | N = 19.0 (24.2%) | N = 2.6 (21.1%) | N = 1.5 (22.5%) | N = 2.7 (17.1%) |
| Expected LLT utilization – 2018 US guideline^†^ | 47.2% | 53.4% | 42.1% | 42.9% |
| Expected LLT utilization – 2022 US ACC pathway^†^ | 47.2% | 53.4% | 42.1% | 42.9% |
| Expected LLT utilization – 2018 2019 EU guideline^†^ | 88.1% | 81.9% | 84.1% | 85.0% |
| **Secondary Prevention**^‡^ | N = 10.0 | N = 1.9 | N = 0.4 | N = 1.3 |
| Observed LLT utilization in NHANES | N = 7.4 (73.5%) | N = 1.0 (52.6%) | N = 0.3 (65.4%) | N = 0.6 (45.4%) |
| Expected LLT utilization – 2018 US guideline^†^ | 100% | 100% | 100% | 100% |
| Expected LLT utilization – 2022 US ACC pathway^†^ | 100% | 100% | 100% | 100% |
| Expected LLT utilization – 2018 2019 EU guideline^†^ | 100% | 100% | 100% | 100% |

*N is in millions

^†^Expected guideline-concordant utilization is defined as the proportion of US patients who would be prescribed each drug class under ideal conditions of fully guideline- and pathway-concordant care.

^‡^Secondary prevention is defined as individuals with clinical cardiovascular disease for both the US guidelines and EU guidelines. Clinical cardiovascular disease was defined as individuals in NHANES who self-reported coronary heart disease, angina/angina pectoris, myocardial infarction, or stroke.

**LLT** lipid lowering therapy

**Source:** National Health and Nutrition Examination Survey, 2013-March 2020

**eFigure 1.** Detailed flowchart depicting assignment to LLT by the 2018 US guideline.

**eFigure 2.** Detailed flowchart depicting assignment to LLT by the 2019 EU guideline.

**eFigure 3.** Detailed flowchart depicting assignment to LLT by the 2022 US pathway.

**Conflict of Interest Statement**

Dr. Alexander is past Chair of FDA’s Peripheral and Central Nervous System Advisory Committee and is a co-founding Principal and equity holder in Stage Analytics. Outside of this work, Dr. Martin has received personal consulting fees from Amgen, AstraZeneca, BMS, Kaneka, Merck, NewAmsterdam, Novartis, Novo Nordisk, Premier, Sanofi, and 89bio. Outside of this work, Dr. Michos has received personal consulting fees from Amgen, Arrowhead, AstraZeneca, Boehringer Ingelheim, Edwards Lifescience, Esperion, Ionis, Lilly, Medtronic, Merck, NewAmsterdam, Novartis, Novo Nordisk, and Pfizer. These arrangements have been reviewed and approved by Johns Hopkins University in accordance with its conflict of interest policies. Dr. Victores, Dr. Bash and Dr. Exter are employees of Merck Sharp & Dohme LLC, a subsidiary of Merck & Co., Inc., Rahway, NJ, USA. Ms. Curran is now employed by Boehringer Ingelheim.

**REFERENCES**

1. Conroy RM, Pyörälä K, Fitzgerald AP, et al. Estimation of ten-year risk of fatal cardiovascular disease in Europe: the SCORE project. Eur Heart J. 2003;24:987-1003.
2. SCORE2 working group and ESC Cardiovascular risk collaboration. SCORE2 risk prediction algorithms: new models to estimate 10-year risk of cardiovascular disease in Europe. Eur Heart J. 2021;42:2439-2454.
3. Heart Disease FastStats. U.S. Centers for Disease Control and Prevention. Available at: https://www.cdc.gov/nchs/fastats/heart-disease.htm (Accessed June 15, 2023).
4. Gao Y, Shah LM, Ding J, Martin SS. US Trends in Cholesterol Screening, Lipid Levels, and Lipid-Lowering Medication Use in US Adults, 1999 to 2018. J Am Heart Assoc. 2023;12:e028205.
5. Olufade T, Zhou S, Anzalone D, Kern DM, Tunceli O, Cziraky MJ, Willey VJ. Initiation Patterns of Statins in the 2 Years After Release of the 2013 American College of Cardiology/American Heart Association (ACC/AHA) Cholesterol Management Guideline in a Large US Health Plan. J Am Heart Assoc. 2017;6:e005205.
6. Gao Y, Shah LM, Ding J, Martin SS. US Trends in Cholesterol Screening, Lipid Levels, and Lipid-Lowering Medication Use in US Adults, 1999 to 2018. J Am Heart Assoc. 2023;12:e028205.
7. Lloyd-Jones D, Morris P, et al. 2022 ACC Expert Consensus Decision Pathway on the Role of Nonstatin Therapies for LDL-Cholesterol Lowering in the Management of Atherosclerotic Cardiovascular Disease Risk. *J Am Coll Cardiol.* 2022;80:1366–1418.
8. McKinley EC, Bittner VA, Brown TM, Chen L, Exter J, Farkouh ME, Huang L, Jackson EA, Levitan EB, Orroth KK, Reading SR, Rosenson RS, Safford MM, Woodward M, Muntner P, Colantonio LD. The Projected Impact of Population-Wide Achievement of LDL Cholesterol <70 mg/dL on the Number of Recurrent Events Among US Adults with ASCVD. Cardiovasc Drugs Ther. 2023 Feb;37(1):107-116. doi: 10.1007/s10557-021-07268-x. Epub 2021 Oct 2. PMID: 34599698.
9. Grundy SM, Stone NJ, Bailey AL, et al. 2018 AHA/ACC/AACVPR/AAPA/ABC/ACPM/ADA/AGS/APhA/ASPC/NLA/PCNA Guideline on the Management of Blood Cholesterol: Executive Summary: A Report of the American College of Cardiology/American Heart Association Task Force on Clinical Practice Guidelines J Am Coll Cardiol. 2019;73:3168-3209.
10. McKinley EC, Bittner VA, Brown TM, Chen L, Exter J, Farkouh ME, Huang L, Jackson EA, Levitan EB, Orroth KK, Reading SR, Rosenson RS, Safford MM, Woodward M, Muntner P, Colantonio LD. The Projected Impact of Population-Wide Achievement of LDL Cholesterol <70 mg/dL on the Number of Recurrent Events Among US Adults with ASCVD. Cardiovasc Drugs Ther. 2023 Feb;37(1):107-116. doi: 10.1007/s10557-021-07268-x. Epub 2021 Oct 2. PMID: 34599698.
11. Grundy SM, Stone NJ, Bailey AL, et al. 2018 AHA/ACC/AACVPR/AAPA/ABC/ACPM/ADA/AGS/APhA/ASPC/NLA/PCNA Guideline on the Management of Blood Cholesterol: Executive Summary: A Report of the American College of Cardiology/American Heart Association Task Force on Clinical Practice Guidelines J Am Coll Cardiol. 2019;73:3168-3209.
12. McKinley EC, Bittner VA, Brown TM, Chen L, Exter J, Farkouh ME, Huang L, Jackson EA, Levitan EB, Orroth KK, Reading SR, Rosenson RS, Safford MM, Woodward M, Muntner P, Colantonio LD. The Projected Impact of Population-Wide Achievement of LDL Cholesterol <70 mg/dL on the Number of Recurrent Events Among US Adults with ASCVD. Cardiovasc Drugs Ther. 2023 Feb;37(1):107-116. doi: 10.1007/s10557-021-07268-x. Epub 2021 Oct 2. PMID: 34599698.
13. Grundy SM, Stone NJ, Bailey AL, et al. 2018 AHA/ACC/AACVPR/AAPA/ABC/ACPM/ADA/AGS/APhA/ASPC/NLA/PCNA Guideline on the Management of Blood Cholesterol: Executive Summary: A Report of the American College of Cardiology/American Heart Association Task Force on Clinical Practice Guidelines J Am Coll Cardiol. 2019;73:3168-3209.
14. Mach F, Baigent C, Catapano AL, et al. 2019 ESC/EAS Guidelines for the management of dyslipidaemias: lipid modification to reduce cardiovascular risk. Eur Heart J. 2020;41:111-188.
